# Multi-electron chemistry for high-energy-density aqueous organic redox flow batteries

**DOI:** 10.1093/nsr/nwag576

**Published:** 2026-09-07

**Authors:** Jiafeng Lei, Yi-Chun Lu

**Affiliations:** Electrochemical Energy and Interfaces Laboratory, Department of Mechanical and Automation Engineering, The Chinese University of Hong Kong, China; Electrochemical Energy and Interfaces Laboratory, Department of Mechanical and Automation Engineering, The Chinese University of Hong Kong, China

Redox flow batteries (RFBs) are promising technologies for large-scale energy storage owing to their high stability and long service life [1]. Although all-vanadium RFBs have been commercially deployed for decades, their broader application is constrained by raw-material price fluctuations and the limited abundance of vanadium. Organic redox-active materials are therefore particularly attractive because of their structural tunability, potentially low cost and reliance on earth-abundant elements such as C, H, O, N and S [2–5]. Through molecular engineering and functional-group modification, their redox potential, solubility, kinetics and stability can be precisely tuned. A wide variety of organic molecules, including quinones, phenazines, viologens and isoalloxazines, have been explored for RFBs [2,4,6]. However, most undergo only one- or two-electron redox processes and often suffer from limited stability at high concentrations (electron concentration >2 M), restricting the practical energy density. The energy density of the electrolyte in RFBs can be expressed as Equation (1):


(1) \begin{eqnarray*}
{\mathrm{Energy\ density}} = \frac{{n\ \times \Delta E\ \times \ c\ \times \ F}}{{3600}},
\end{eqnarray*}

where *n* is the number of electrons transferred per molecule, *Δ **E* is the potential difference between the positive and negative redox couples, *c* is the concentration of active materials and *F* is the Faraday constant. Thus, the common strategies to improve energy density (Fig. 1a) include increasing the concentration of active materials (increase *c*) and manipulating the redox potential of active materials to increase the full cell voltage (increase *Δ **E*). Increasing the number of electrons is always challenging for specific molecules with limited active centers, and previous efforts toward multi-electron transfer have often shown poor stability at high concentrations [7,8].

**Figure 1.**
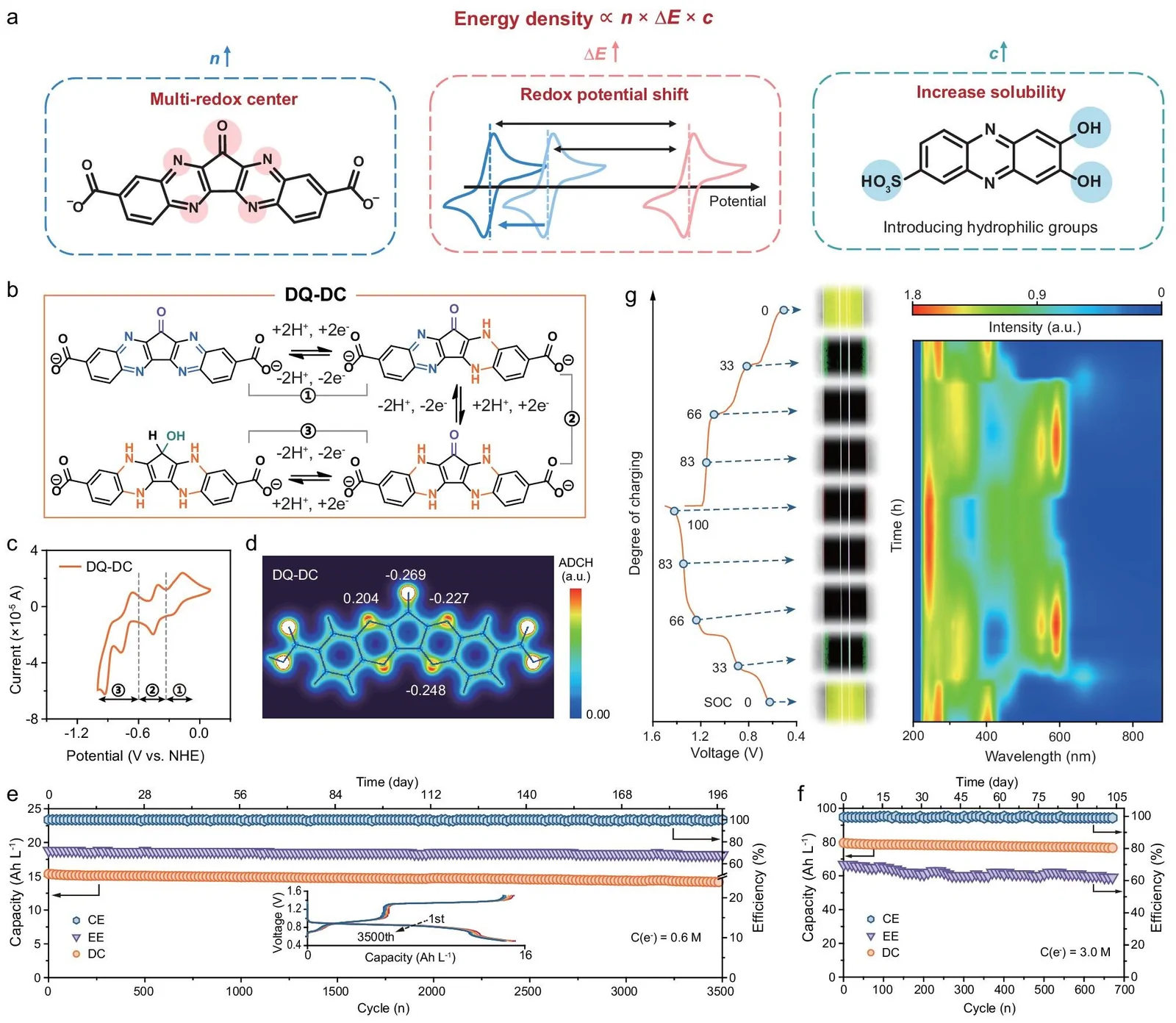
(a) The common strategies for increasing the energy density of organic-based RFBs. (b) The proposed reaction mechanism of DQ-DC molecules. (c) The CV profile of the DQ-DC molecule. (d) The calculated ADCH atomic charge values. The long-term cycling stability of DQ-DC at an electron concentration of (e) 0.6 M and (f) 3 M. (g) The optical images of the negolyte at different SOCs and the corresponding operando UV-vis spectra. Adapted with permission from [9].

Recently, writing in *National Science Review*, Song, Kong and co-workers reported a molecular engineering strategy that integrates phenazine and ketone redox centers within a single *π *-conjugated framework, enabling a highly reversible six-electron redox process [9]. This design enables multiple redox centers to operate sequentially and cooperatively within one molecule, providing a new strategy for multi-electron organic molecules. Unlike simply increasing the number of redox-active sites, this approach focuses on coordinating their electronic interactions to achieve efficient multi-electron storage. To enhance aqueous solubility under alkaline conditions, carboxylic acid groups were introduced into the molecular scaffold, yielding the redox-active phenazine–ketone molecule, designated DQ-DC (Fig. 1b). Cyclic voltammetry revealed four well-defined redox couples, suggesting a reversible multi-step electron-transfer process (Fig. 1c). To elucidate this multi-electron redox process, a combination of electrochemical analysis and *ex situ* nuclear magnetic resonance (NMR), electron paramagnetic resonance (EPR) and Raman spectroscopy was used to track the structural evolution of DQ-DC during charging and discharging. The complementary spectroscopic results reveal a sequential reduction pathway in which the phenazine units are reduced first, followed by reduction of the fluorenone carbonyl (Fig. 1b). The reverse process occurs during discharge. Together with theoretical calculations, these results demonstrate that the conjugated framework enables the two redox-active motifs to operate cooperatively and reversibly, giving rise to six-electron storage within a single molecule. Compared with Q-C, which contains only one pyrazine moiety, and the fluorenone-based control compound FL-DC, DQ-DC exhibits substantially higher volumetric capacity and significantly improved cycling stability. A potential concern associated with fusing multiple redox centers is that the resulting increase in molecular size may compromise mass transport and electron-transfer kinetics. However, DQ-DC still maintains favorable transport properties, with diffusion coefficients *D*_0_ of 3.81 *× * 10^−5^, 1.40 *× * 10^−5^ and 2.17 *× * 10^−6^ cm^2^ s^−1^ for its three major redox processes. The corresponding heterogeneous electron-transfer rate constants *k*_0_ reach 3.66 *× * 10^−2^, 2.65 *× * 10^−3^ and 1.36 *× * 10^−^^3^ cm s^−1^, respectively, demonstrating that the fused molecular architecture retains fast kinetics. Moreover, DQ-DC achieves a high solubility of 0.57 M in 1 M KOH electrolyte, corresponding to a high theoretical volumetric capacity of approximately 91.6 Ah L^−1^_negolyte_.

Theoretical calculations were further employed to elucidate how heteroaromatic fusion regulates the electronic structure of DQ-DC. Atomic dipole moment-corrected Hirshfeld (ADCH) charge analysis revealed reduced charge polarization of the carbonyl group and a redistribution of negative charge over the pyrazine moieties compared with Q-C and FL-DC, consistent with enhanced π-electron delocalization across the fused framework (Fig. 1d). Combined anisotropy of the induced current density and nucleus-independent chemical shift (NICS) analyses further revealed enhanced aromatic stabilization and reduced antiaromaticity of the fluorenone core. This favorable electronic delocalization is proposed to contribute to higher chemical stability. The DQ-DC-based RFB exhibits a combination of high charge-storage capacity, power output and cycling stability. The system achieves a high volumetric capacity of 79.3 Ah L^−1^_negolyte_ and a peak power density of 250.2 mW cm^−2^. At an electron concentration of 0.6 M, the battery operates for 3500 cycles (198.5 days) with a low decay rate of 0.039% per day (Fig. 1e). Even at 3 M electron concentration, stable cycling can still be maintained, demonstrating a favorable balance among electron-transfer number, capacity, power capability and long-term stability (Fig. 1f). This achievement not only benchmarks the durability of concentrated multi-electron electrolytes, but also establishes hetero-redox-center fusion as a promising molecular design principle for pushing organic RFBs toward higher energy densities. Interestingly, DQ-DC exhibits distinct and reversible color changes from yellow to green, purple and ultimately dark red with increasing state of charge (SOC). Together with the characteristic changes in its ultraviolet–visible (UV-vis) spectrum, this intrinsic chromatic response offers a simple route for visual or spectroscopic SOC monitoring (Fig. 1g).

Despite the substantial progress demonstrated by this molecular design strategy, efforts are still required to advance its practical implementation. First, future molecular engineering could explore the integration of additional redox-active centers to further increase the electron-transfer number per molecule. In parallel, functional-group modification may improve aqueous solubility and thereby further increase the accessible volumetric capacity. Second, current fused multi-redox designs are predominantly developed around quinone-, phenazine- and fluorenone-based motifs for negolyte applications. However, the limited availability of high-capacity and high-potential posolytes remains another major bottleneck constraining full-cell energy density. Extending multi-redox-center design concepts to the posolyte side is therefore highly desirable. Third, the increasingly complex multi-electron chemistry calls for more advanced characterization techniques to resolve individual electron-transfer steps, identify transient intermediates and clarify the origin of multiple voltage plateaus [10]. Such mechanistic insights will be essential for establishing structure–performance relationships and guiding the rational design of high-energy-density organic molecules.

In summary, this work developed a molecular fusion strategy that integrates multiple heterogeneous redox centers into a single phenazine–ketone scaffold, enabling a reversible six-electron redox process. The resulting multi-redox molecule combines favorable reaction kinetics with significantly improved stability, delivering a high volumetric negolyte capacity of over 79 Ah L^−1^_negolyte_ for more than 108 days of operation. By integrating distinct redox-active motifs and tuning their electronic coupling, this strategy offers a general route to constructing cooperative multi-redox organic molecules. Beyond the phenazine–fluorenone system, this concept may be extended to other redox motifs to enable multi-electron organic molecules with high concentration and long-term stability.
